# Is there a relationship between the prevalence of autoimmune thyroid disease and diabetic kidney disease?

**DOI:** 10.1515/biol-2021-0064

**Published:** 2021-06-21

**Authors:** Magdalena Maria Stefanowicz-Rutkowska, Wojciech Matuszewski, Katarzyna Gontarz-Nowak, Elżbieta Maria Bandurska-Stankiewicz

**Affiliations:** Clinic of Endocrinology, Diabetology and Internal Diseases, School of Medicine, Collegium Medicum, University of Warmia and Mazury in Olsztyn, ul. Żołnierska 18 (Wojewódzki Szpital Specjalistyczny w Olsztynie, pok. 32), 10-957 Olsztyn, Poland

**Keywords:** diabetes, autoimmune thyroid disease, diabetic kidney disease, albuminuria

## Abstract

Autoimmune thyroid disease (AITD) is more common among diabetes mellitus (DM) patients and may impact its microvascular complications. The present study aimed to assess the relationship between AITD and the prevalence of diabetic kidney disease (DKD) in patients with diabetes mellitus type 1 (DM1). Anthropometric parameters, parameters of metabolic control of DM, thyreometabolic status, and the UACR were assessed. DKD was diagnosed if patients’ UACR level was ≥30 mg/g or eGFR level was <60 mL/min. This study involved 144 patients with DM1 aged 36.2 ± 11.7 years: 49 men and 95 women. Significant differences in creatinine, eGFR, and UACR levels were found in patients with DKD. fT3 concentration was significantly lower among DKD patients. A significantly higher probability of DKD was found in DM1 patients with lower fT3 levels. Patients with DM1 and AITD had significantly lower creatinine levels than the control group. However, the study did not show any significant relationship between AITD and the occurrence of DKD in patients with DM1. Significantly lower fT3 concentrations in DKD patients may be caused by metabolic disorders in the course of DKD and require further cohort studies in a larger population of patients with DM1 and AITD.

## 1 Introduction

Diabetes mellitus (DM) is a chronic, lifelong progressive metabolic disease characterized by hyperglycemia due to absolute or relative insulinopenia. DM might be called the epidemic of the present age and is caused by a complex interplay between genetic predisposition and environmental factors [1,2,3]. Diabetic kidney disease (DKD) is one of the major chronic complications in diabetes mellitus type 1 (DM1) and 2 and is currently the most common cause of the end-stage renal disease (ESRD). DM contributes to approximately 40% of the newly diagnosed ESRD cases in patients each year [4,5]. The pathogenesis of DKD is multifactorial. Moreover, hemodynamic and metabolic changes may affect the progression of DKD [6,7]. The incidence of thyroid disease is higher in patients with DM [8,9]. DM1 and autoimmune thyroid disease (AITD) combine similar pathogenetic mechanisms leading to the synthesis of autoantibodies directed against the cellular elements of endocrine organs and body tissues, causing tissue infiltration from immunocompetent cells, destruction dependent on the immune system, and organ-specific humoral response [10,11,12]. The present observation is a continuation of our previous research, where we showed that the coexistence of AITD in patients with DM1 is associated with a significantly lower risk of developing nonproliferative diabetic retinopathy (NPDR) [13]. AITD and DKD are associated with endothelial dysfunction [14,15], yet the coexistence of AITD and DKD has not yet been assessed. The aim of the study is to assess the relationship between AITD and the occurrence of DKD in a group of patients with DM1.

## 2 Materials and methods

### 2.1 Clinical assessment

The retrospective, cross-sectional and noninterventional study was performed on a group of patients with DM1 diagnosed according to World Health Organization (WHO) criteria. The clinical research center for this particular investigation was the Department of Endocrinology, Diabetology and Internal Diseases in Olsztyn. Medical records of hospitalized patients between 2015 and 2020 were analyzed. The study included adult patients with DM1 lasting over a year and AITD lasting more than a year diagnosed based on a positive antithyroglobulin antibody (ATA) titer and ultrasound images of the thyroid gland. The study excluded patients with genetically determined diseases, refractory hypertension, systemic diseases, NYHA stage III and IV heart failure, chronic respiratory diseases, liver failure, eGFR kidney failure <30 mL/min, tumors, mental illness, alcohol abuse, and/or drug addiction. The control group consisted of patients without AITD appropriately matched for age, BMI, diabetes duration, and metabolic control. Methodologies used in diagnosing DM metabolic control, thyroid metabolism, AITD, and DR are as follows: clinical physical examination, blood pressure (BP) assessment, laboratory tests including biochemical and hormonal concentrations, and statistics. Patient data about age, DM1 duration, and history of hypertension and/or dyslipidemia were also obtained. For each patient, height and weight and systolic and diastolic BP (mm Hg) were also examined. The Laboratory of the Provincial Hospital in Olsztyn was an experimental research center, where the following biochemical and hormonal concentrations were evaluated in serum: glycated hemoglobin (HbA1c), total cholesterol (TC), high-density lipoprotein (HDL – cholesterol), low-density lipoprotein (LDL – cholesterol), triglyceride (TG), and creatinine with the evaluation of the estimated glomerular filtration rate (eGFR), calculated using the CKD-EPI formula. Urinary albumin excretion was measured as the albumin-to-creatinine ratio (UACR) in the morning urine sample. Albumin excretion of ≥30 and <300 mg per gram of creatinine in the urine was considered microalbuminuria, and ≥300 as macroalbuminuria. DKD was diagnosed with UACR ≥ 30 mg/g in the absence of other renal abnormalities or eGFR < 60 mL/min/1.73 m^2^, according to the KDOQI recommendations from 2012 [16]. Thyroid function was assessed by examining free thyroxine (fT4), free triiodothyronine (fT3), and pituitary hormone–thyroid-stimulating hormone (TSH), as well as ATA titers: antithyroid peroxidase antibodies (aTPO), antithyroglobulin antibodies (aTG), and TSH receptor autoantibodies (anti-TSHR, TRAb). Among patients, thyroid gland ultrasounds were also performed.


**Informed consent:** Informed consent has been obtained from all individuals included in this study.
**Ethical approval:** The research related to human use has been complied with all the relevant national regulations, institutional policies and in accordance with the tenets of the Helsinki Declaration, and has been approved by the Bioethical Committee of School of Medicine, University of Warmia and Mazury in Olsztyn, Poland.

### 2.2 Measurement methodology

#### 2.2.1 Physical examination

Physical examination was performed in both examined groups of patients, taking into account anthropometric measurements and measurements of BP. BP was measured twice using the Korotkoff method in a sitting position after a 10 min rest. Hypertension was diagnosed if systolic blood pressure (SBP) was ≥140 mm Hg and diastolic blood pressure (DBP) was ≥90 mm Hg or the patient had already been treated with antihypertensive drugs.

#### 2.2.2 Biochemical parameters and hormonal concentrations

Biochemical and hormonal concentrations tests were performed at the Laboratory of the Provincial Hospital in Olsztyn according to standard analytical procedures and quality assurance protocols in force. Venous blood was collected using vacuum/suction sets from the basilic vein, in the morning, in fasting – after a 12 h break after eating and drinking. Immediately after transfer to the tubes and clot formation, blood was centrifuged at +4°C. Biochemical and hormonal parameters were evaluated in serum by enzymatic-calorimetric and electrochemiluminescence methods using a Roche chemistry analyzer Cobas 6000/c501.

The results included HbA1c, TC, (HDL – cholesterol), low-density lipoprotein, (LDL – cholesterol), TG, and creatinine with the evaluation of eGFR calculated on the basis of creatinine concentration according to the CKD-EPI formula.

#### 2.2.3 Thyroid function

Thyroid function was assessed by examining thyroid hormone levels: fT4 and fT3 and pituitary hormone–TSH, as well as ATA titers, including aTPOs, aTGs, and thyroid-stimulating hormone receptor antibodies (TRAbs).

### 2.3 Statistical analysis

Continuous variables were expressed as mean and standard deviation (mean ± SD). Qualitative data are presented as structure indicators (%). Normality was calculated using the Shapiro–Wilk test. Significance in the difference between the two groups was tested by Student’s *t*-test when the normality criteria were met and by the Mann–Whitney *U* test when they were not met. Logistic regression analysis was used to analyze the association between AITD and DKD. The odds ratio (OR) and 95% confidence interval were calculated utilizing logistic regression analysis. The level of significance was set at *α* = 0.05. The data were analyzed using the statistics software Statistica 13.3 PL program for Windows.

## 3 Results

The following research involved medical records of 144 patients aged 36.2 ± 11.7 years: 49 (34%) men and 95 (66%) women. The mean duration of DM1 in the whole group was 13.32 ± 9.9 years, while SBP was 116.9 ± 12 mm Hg, DBP was 76.4 ± 9.8, and the HbA1c rate was 8.6 ± 1.68%. Renal parameters in the whole group were as follows: creatinine 0.78 ± 0.2 mg/dL, eGFR 109.32 ± 22.48 mL/min/1.73 m^2^, and UACR 2.2 ± 5.7 mg/g. Thyroid function indices were TSH 2.3 ± 4.1 mIU/L, fT3 4.53 ± 0.93 pmol/L, fT4 16.56 ± 3.28 pmol/L, a-TPO 109.81 ± 159.78 IU/mL, and a-TG 105.1 ± 206.47 IU/mL.

The study group consisted of 68 patients with DM1 and AITD, aged 35 ± 11.4 years, of whom 62 (91%) were women and 6 (9%) men. The control group consisted of 76 patients with DM1 and without AITD, aged 37.2 ± 11.9 years, of whom 33 (43%) were women and 43 (57%) were men. They were selected according to age, BMI, diabetes duration, and metabolic control. The mean BMI was 24.1 ± 4.2 kg/m^2^ in the study group and 23.7 ± 3.3 kg/m^2^ in the control group. The mean duration of DM1 was 12.4 ± 10.5 years in the study group and 14.2 ± 9.3 years in the control group. The metabolic control parameters were as follows: SBP, 116.1 ± 12.8; DBP, 75.4 ± 10 mm Hg; and HbA1c, 8.3 ± 1.8% in the study group and SBP, 117.5 ± 11.3 mm Hg; DBP, 77.3 ± 9.6 mm Hg; and HbA1c, 8.8 ± 1.6% in the control group. Renal parameters in the study group stood at the following levels: creatinine, 0.7 ± 0.2 mg/dL; eGFR, 111.54 ± 23.2 mL/min/1.73 m^2^; UACR, 1.7 ± 3.4 mg/g compared to the control group (creatinine, 0.8 ± 0.2 mg/dL; eGFR, 107.32 ± 21.8 mL/min/1.73 m^2^; UACR, 2.6 ± 7.2 mg/g). Thyroid function indices were as follows: TSH, 2.76 ± 5.8 mIU/L; fT3, 4.46 ± 1.1 pmol/L; fT4, 17.02 ± 4 pmol/L; a-TPO 216.21 ± 180.8 IU/mL; and a-TG, 204.46 ± 268 IU/mL in the study group and TSH, 1.9 ± 0.9 mIU/L; fT3, 4.59 ± 0.8 pmol/L; fT4, 16.14 ± 2.4 pmol/L; a-TPO, 14.61 ± 6.3 IU/mL; and a-TG 16.2 ± 13.4 IU/mL in the control group. There was a significantly lower concentration of creatinine and a significantly higher concentration of anti-TPO and anti-Tg in the test group versus the control group. The obtained data are presented in Tables 1 and 2.

**Table 1:** Patient characteristics (*n* = 144)

Patient characteristics	Mean ± SD
Men (%)	34
Age (years)	36.2 ± 11.7
Body mass index (kg/m^2^)	23.9 ± 3.7
Duration of DM1 (years)	13.32 ± 9.9
SBP (mm Hg)	116.9 ± 12
DBP (mm Hg)	76.4 ± 9.8
HbA1c (%)	8.6 ± 1.68
TC (mg/dL)	179.7 ± 45.1
TG (mg/dL)	102.8 ± 55.8
HDL (mg/dL)	70.2 ± 21.5
LDL (mg/dL)	101.7 ± 33.8
Creatinine (mg/dL)	0.78 ± 0.2
eGFR (mL/min/1.73 m^2^)	109.32 ± 22.48
UACR (mg/g)	2.2 ± 5.7
TSH (mIU/L)	2.3 ± 4.1
fT3 (pmol/L)	4.53 ± 0.93
fT4 (pmol/L)	16.56 ± 3.28
a-TPO (IU/mL)	109.81 ± 159.78
a-TG (IU/mL)	105.1 ± 206.47

**Table 2:** The differences between patients with and without AITD

Parameters	No-AITD (*n* = 76)	AITD (*n* = 68)	*p*
Men (%)/women (%)	56.6/43.4	8.8/91.2	<0.01
Age (years)	37.2 ± 11.9	35 ± 11.4	0.25
Duration of DM1 (years)	14.2 ± 9.3	12.4 ± 10.5	0.28
Body mass index (kg/m^2^)	23.7 ± 3.3	24.1 ± 4.2	0.54
SBP (mm Hg)	117.5 ± 11.3	116.1 ± 12.8	0.27*
DBP (mm Hg)	77.3 ± 9.6	75.4 ± 10	0.11*
HbA1c (%)	8.8 ± 1.6	8.3 ± 1.8	0.07
TC (mg/dL)	177.5 ± 46.3	182.1 ± 43.9	0.55
HDL (mg/dL)	67.6 ± 23.5	73 ± 18.7	0.09*
LDL (mg/dL)	98.7 ± 30.2	105 ± 37.3	0.27
TG (mg/dL)	110.5 ± 59.1	94.2 ± 51.1	0.08
Creatinine (mg/dL)	0.8 ± 0.2	0.7 ± 0.2	<0.01
eGFR (mL/min/1.73 m^2^)	107.32 ± 21.8	111.54 ± 23.2	0.26
UACR (mg/g)	2.6 ± 7.2	1.7 ± 3.4	0.35
TSH (mIU/L)	1.9 ± 0.9	2.76 ± 5.8	0.20
fT3 (pmol/L)	4.59 ± 0.8	4.46 ± 1.1	0.40
fT4 (pmol/L)	16.14 ± 2.4	17.02 ± 4	0.11
a-TPO (IU/mL)	14.61 ± 6.3	216.21 ± 180.8	<0.001
a-TG (IU/mL)	16.2 ± 13.4	204.46 ± 268	<0.001

*Mann–Whitney *U* test.

The incidence of DKD among patients with DM1 was 3.5%. Significant differences in the concentration of creatinine, eGFR, and UACR were found in patients with and without DKD. fT3 concentration was significantly lower among DKD patients. ATA concentration and other variables did not differ significantly between the two groups. The differences between patients without DKD and with DKD are presented in Table 3.

**Table 3:** Comparison of patients with and without DKD

Parameters	No-DKD (*n* = 139)	DKD (*n* = 5)	*p*
Men (%)/women (%)	33.33/63.2	0.69/2.78	0.50
Age (years)	36.1 ± 11.8	39.2 ± 7.3	0.56
Duration of DM1 (years)	13.1 ± 9.9	18.2 ± 9	0.26
Body mass index (kg/m^2^)	23.9 ± 3.8	25.2 ± 3.4	0.43
SBP (mm Hg)	116.7 ± 12	120.8 ± 13	0.58*
DBP (mm Hg)	76.1 ± 9.8	84 ± 8	0.10*
HbA1c (%)	8.6 ± 1.7	8,8 ± 1.6	0.78
TC (mg/dL)	178.9 ± 42.5	200.6 ± 98.2	0.29
TG (mg/dL)	102 ± 54.2	125.4 ± 96.6	0.36
HDL (mg/dL)	70.5 ± 21.5	60.4 ± 22.3	0.48*
LDL (mg/dL)	102.1 ± 34.1	89.4 ± 20.9	0.41
Creatinine (mg/dL)	0.8 ± 0.2	1.1 ± 0.4	<0.001
eGFR (mL/min/1.73 m^2^)	110.6 ± 21.2	74.2 ± 30.4	<0.001
UACR (mg/g)	1.5 ± 2.8	21.4 ± 20.3	<0.001
TSH (mIU/L)	2.33 ± 4.1	1.84 ± 0.8	0.79
fT3 (pmol/L)	4.57 ± 0.9	3.36 ± 0.6	<0.01
fT4 (pmol/L)	16.6 ± 3.3	15.38 ± 3.7	0.42
a-TPO (IU/mL)	110.68 ± 160.3	85.4 ± 160.2	0.73
a-TG (IU/mL)	107.76 ± 209.6	31 ± 31.4	0.42

*Mann–Whitney *U* test.

**Figure 1:**
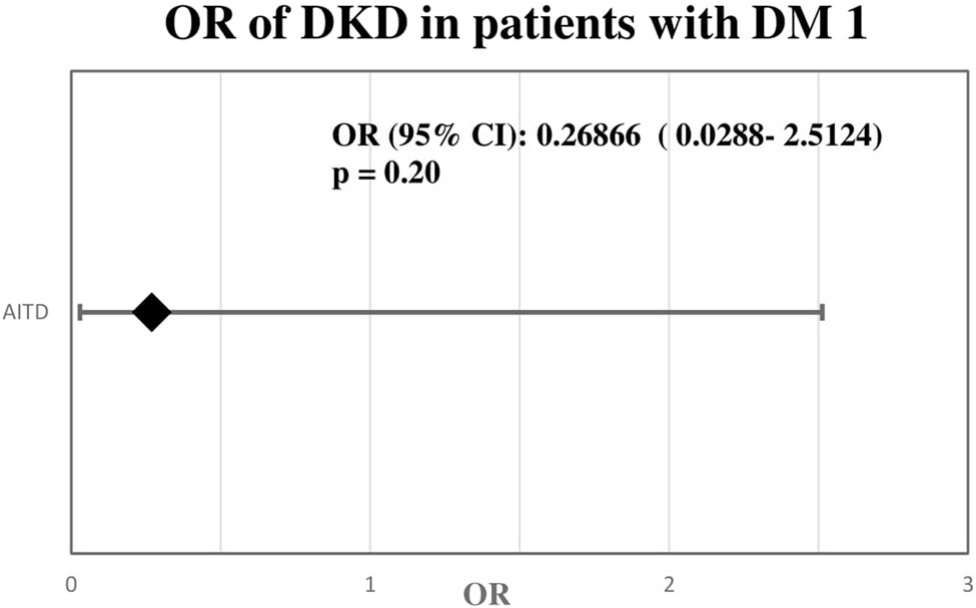
Odds ratio of diabetic kidney disease in patients with diabetes mellitus type 1 and autoimmune thyroid disease and in the group with DM1 without AITD.

There was no significant difference in the prevalence of DKD among DM1 patients with AITD and the control group in logistic regression analysis. The correlations are presented in Figure 1.

**Figure 2:**
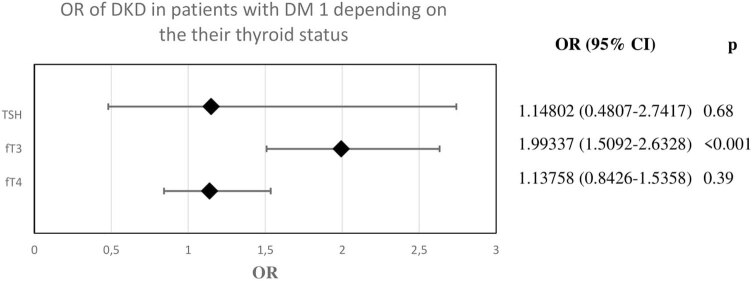
Odds ratio of diabetic kidney disease in patients with diabetes mellitus type 1 depending on their thyroid status.

A significantly higher probability of DKD was found in patients with DM1 who had lower fT3 levels (Figure 2).

## 4 Discussion

### 4.1 AITD and DM1

The study was conducted to assess the correlation between the occurrence of AITD and the development of DKD. AITD and DM1 are common autoimmune diseases [17]. AITD occurs in 17–30% of DM1 patients, and approximately 25% of children with DM1 have antithyroid antibodies (ATA) at diagnosis. Their presence is a predictor of thyroid dysfunction, most often hypothyroidism [18]. Due to the fact that the assessment of thyroid function may be unreliable if it is performed at the time of diagnosis of DM due to previous hyperglycemia, ketosis, ketoacidosis, and weight loss, in our study, thyroid function was assessed at least 1 year after the diagnosis of DM1 during the period of metabolic stabilization and good glycemic control. The prevalence of ATA ranges from 1 to 4% in the general population and from 3 to 50% in children with DM1 in different countries and populations [19]. ATA appears within 2.5–3 years of disease progression [20]. Studies assessing the presence of ATA in patients with DM1 differ significantly regarding the incidence of AITD, as presented in Table 4 [21,22,23,24].

**Table 4:** The incidence of AITD in patients with DM1

Study	Number of patients (*n*)	Incidence of AITD (%)
Lee et al.	139	38.8
Kang et al.	59	28.8
Jung et al.	73	26
Hwang et al.	102	30.4

Multiple studies have shown that age, female gender, disease duration, and the presence of beta-cell autoimmunity are risk factors for developing AITD in patients with DM1. In studies by Hwang et al. and Jin et al., the presence of antiglutamic acid decarboxylase antibodies (GADAs) at the time of DM1 diagnosis was a significant predictor of AITD development [24,25]. However, the exact mechanism by which GADA influences thyroid autoimmunity is unclear. Jung et al. proved that the presence of insulin autoantibodies at the initial DM1 diagnosis was correlated with a positive ATA result [23]. Moreover, it has been proven that DM1 and AITD have a common genetic basis [17,26,27]. It has been observed that AITD is more common in women than in men with DM1 [28,29]. Androgens may have a protective effect against autoimmunity, while estradiol appears to accelerate the progression of AITD via the T cell pathway [30]. This is also confirmed by our study, where women constituted 91.2% of patients in the study group.

### 4.2 Thyroid dysfunction in patients with chronic kidney disease (CKD)

Thyroid dysfunction can cause changes in renal blood flow, glomerular filtration, absorption and secretion of renal tubules, electrolyte homeostasis, and renal structure [31]. In patients with CKD, most commonly, decreased concentration of T3 is observed, which is associated with a decrease in the peripheral T4 to T3 conversion. Chronic metabolic acidosis associated with CKD may contribute to this [31,32]. Zoccali et al. observed that low fT3 concentration is common in inflammatory diseases and in CKD and ESRD patients and also observed that fT3 is acutely and reversibly inhibited in CKD patients during inflammatory processes caused by coexisting infections [33]. In their cohort study, Zhang et al. noted that low fT3 levels were associated with an increased risk of CKD in the euthyroid patient population [34]. AITD has not been found to be more common in CKD patients. In fact, the incidence of ATA is low in CKD patients. However, AITD can occur together with other autoimmune diseases associated with CKD, such as lupus nephritis and DM1 [35]. Therefore, screening for AITD in these patients is important. The most common kidney diseases seen in AITD are membranous glomerulonephritis, submicroscopic glomerulonephritis, IgA nephropathy, focal segmental glomerulosclerosis, antineutrophil cytoplasmic antibodies (ANCAs), and amyloidosis. The most likely mechanisms of coexistence of AITD with glomerulopathies are the glomerular deposition of immune complexes of thyroglobulin and autoantibodies, as well as impaired immune tolerance to megalin (a glycoprotein regulated by TSH, located on the thyrocyte apical surface) [36].

### 4.3 The pathogenesis, risk factors, and treatment of DKD

The prevalence of DKD increases in parallel with the incidence of DM [37,38]. The pathogenesis of DKD is complex and multifactorial, including genetic and environmental factors. The causes of DKD include nonmodifiable risk factors such as genetic profile, sex, age, age of onset of DM, and duration of DM. However, there are also several modifiable risk factors, including glycemic control, BP, lipid control, and smoking, as well as low-grade chronic inflammation, advanced glycation end-products, and physical inactivity [39]. Although control of glucose and BP should prevent DKD development, many patients with DM progress to ESRD. Additional therapies are required for more effective treatment of DKD. Research is underway on innovative treatment options for DKD, for instance, targeted therapies aiming to halt or prevent complications causing DKD. Several trials using novel drugs targeting the molecular mechanisms of ESRD development have recently been completed or are ongoing. Growing evidence suggests that dipeptidyl peptidase 4 inhibitors and sodium-glucose cotransporter 2 inhibitors exert renoprotective effects [40]. Attempts are made at reducing albuminuria with the use of mineralocorticoid receptor antagonists and endothelin receptor antagonists [40]. Fiorina et al. showed the efficacy of Abatacept (CTLA4-Ig) in B7-1 inhibition on podocytes as a potential therapeutic strategy for the treatment of proteinuria in DKD in patients with podocyte B7-1–positive FSGS [41]. In patients with renal fibrosis, Src family kinases may be a valuable therapeutic target. Taniguchi et al. stated that pharmacological inhibition of Sfc by PP2 and SU6656 blocked high-glucose-stimulated Sfc phosphorylation (at Tyr-416), the EGF receptor (EGFR), mitogen-activated protein kinase, and the tumor necrosis factor α-converting enzyme [42]. Yan et al. showed that PP1-mediated inhibition of the Src kinase reduced fibrosis in either the presence or absence of transforming growth factor-β (TGF-β) stimulation *in vitro* and attenuated renal interstitial fibrosis after the development of unilateral ureteral obstruction *in vivo* [43]. What seems interesting is harnessing the immunological properties of stem cells (SCs) as a therapeutic option for DKD. SCs have anti-inflammatory and immunomodulatory properties. Most of the research concerning SCs is based on animal models (mice). Several studies have emphasized the renoprotective effect of bone marrow mesenchymal stem cells and bone marrow hematopoietic SCs (HSCs) in promoting repair and regeneration of renal structures after injury because of their capacity to be recruited to inflamed or injured areas by local release of chemokines [44]. HSCs have been tested in humans in the treatment of DM1 by rescuing peripheral tolerance toward pancreatic B cells [44]. The infusion of human cord blood mesenchymal stem cells in a murine model of streptozotocin-induced DN resulted in a significant improvement in all compromised parameters, including a reduction in tubular dilatation and glomerular hypertrophy [45]. Currently, there is only one active clinical trial concerning the safety and efficacy of a single intravenous infusion of allogeneic mesenchymal precursor cells (Mesoblast) to be determined in adult patients with DKD and DM2 [44]. The mean duration of DM1 in the whole group was 13.32 ± 9.9 years, and the HbA1c rate was 8.6 ± 1.68%. The mean duration of DM1 in the study group was 12.4 ± 10.5 years and in the control group was 14.2 ± 9.3 years. The control group consisted of patients selected according to age, BMI, diabetes duration, and metabolic control to exclude the recognized risk factors for DKD. To the best of the authors’ knowledge, so far little research has been conducted on the correlation between AITD and DKD in type 2 DM. Microalbuminuria was higher in patients with type 2 DM and prediabetics with subclinical hypothyroidism than in patients with euthyroidism [46]. Further, in a cohort of nearly 30,000 people with type 2 DM, high TSH levels were associated with decreased eGFR [47]. Such observations were even fewer regarding patients with DM1. Rodacki et al. showed that patients with DM1 and TSH levels 0.4–2.5 mU/L had a lower risk of diabetic retinopathy and renal dysfunction. Unfortunately, that study not assessed the ATA [48]. In our study, there was a significantly lower concentration of creatinine and a significantly higher concentration of anti-TPO and anti-Tg in the test group versus the control group. The prevalence of DKD among patients with DM1 was 3.5%.

### 4.4 Relationship between the prevalence of AITD and DKD: Endothelial dysfunction in AITD and DKD

AITD is associated with endothelial dysfunction by impaired production of nitric oxide through the COX-2-dependent pathway, which leads to increased oxidative stress [49]. Chronic inflammation is suggested to be involved in the development of ESRD in DM. Niewczas et al. in their cohort study recognized 17 novel proteins enriched for TNF Receptor superfamily members called kidney risk inflammatory signature (KRIS), which was associated with a 10-year risk of ESRD. KRIS proteins contribute to the inflammatory process underlying ESRD development in both types of DM [50]. Endothelial dysfunction is associated with both AITD and DKD and may be a potential link between the two. Yet, there was no significant difference in the prevalence of DKD among DM1 patients with AITD and in the control group. There were significant differences in the concentration of creatinine, eGFR, and UACR in patients with and without DKD. fT3 concentration was significantly lower among DKD patients. ATA concentration and other variables did not differ significantly between the two groups. A study carried out on a population of middle-aged and elderly Chinese patients showed that low fT3 levels were associated with a higher incidence of microalbuminuria [51]. So far, few studies have been conducted to determine the correlation between fT3 concentration and the incidence of DKD in patients with type 2 DM. They showed that the fT3 concentration was negatively correlated with the incidence of DKD in patients with DM type 2 with normal thyroid function [52,53]. Such studies have not been carried out in the population of DM1 patients so far. Our results were consistent with the aforementioned studies; a significantly higher probability of DKD was shown in patients with DM1 with lower fT3 concentration. Low T3 was closely related to endothelial dysfunction in patients with advanced non-DKD (nondiabetic kidney disease) [54]. Endothelial dysfunction was associated with albuminuria in DM, and hence, low fT3 levels and albuminuria may be associated with endothelial dysfunction [55]. The development of DKD is caused by abnormalities in the enzymatic pathways in which T3 plays an important role. In an animal model with mice, it was shown that T3 increases phosphatidylinositol 3-kinase (PI3K), reduces expression of transforming growth factor β1 (TGF-β1) c, improves structurally damaged kidneys, and reduces albuminuria [56]. 3,5-Diiodothyronine may have a protective effect on kidney cells in DKD by inhibiting activation of nuclear factor kappa-light-chain-enhancer of activated B cells and c-Jun N-terminal kinase [57]. Nevertheless, previous studies of patients with DM2 did not find a relationship between the occurrence of ATA and DKD, which could, however, be associated with a low percentage of positive TPO-Ab in this group (8.0%) [53]. Similarly, our study failed to demonstrate a significant association between AITD and DKD in the DM1 patient population. Potential reasons for this, and at the same time the limitations of the study, are first and foremost the cross-sectional design of the retrospective study without long-term follow-up and the small sample size, which is a major limitation of our results. Second, thyroid function tests and UACR were measured at one time point. The third reason is the predominance of women in our study and the variability of environmental and dietary factors in patients. A prospective study involving a larger number of patients is necessary to determine the correlation between AITD in patients with DM1 and DKD. It is very important to establish standards for the management of concomitant AITD in patients with DM1 to reduce their risk of microangiopathy. In all patients with DM1, regular serologic screening for ATA and evaluation of thyroid function should be considered, even in the absence of symptoms. In addition, if ATA is detected or goiter is diagnosed, the thyroid gland should be examined by ultrasound.

## 5 Conclusion

Patients with DM1 and AITD had significantly lower creatinine levels compared to the control group. However, the present study did not show any significant relationship between AITD and the incidence of DKD in patients with DM1. Significantly lower fT3 concentrations in DKD patients may be caused by metabolic disturbances in the course of this complication and require further cohort studies in a larger population of patients with DM1 and AITD.
